# Invariable Benefit of Mineral Acids in Dropsy

**Published:** 1843-04-01

**Authors:** 


INVARIABLE BENEFIT OF MINERAL ACIDS IN DROPSY.

Dr. Trusen, of Posen, in a memoir in a late number of Hufeland's Journal, states that he has found mineral acids, as recommended by Alix, Meyer, Barez, &c., of universal benefit in dropsy not dependent on disease of the respiratory organs, or extensive disorganization of the liver. The principal medicines of this class employed by M. Trusen are the acid-elixir of Haller and phosphoric acid. The former is useful in dropsies of an adynamic character, those consequent on intermittent fevers, and others due to checks of the perspiration or other secretions. The phosphoric acid is suitable in cases of dropsy owing to an altered condition of the blood, those supervening after diarrhœa, dysentery, chlorosis, &c. He has seldom occasion to give purgatives to patients under the influence of the above medicaments, which of themselves insure a free action of the bowels; but, in some obstinate cases, he has availed himself of the aid of alcoholic vapour-baths to produce a sudorific effect. M. Trusen remarks, in the course of his memoir, that the extensive tumefaction of the scrotum, which presents so marked a tendency to terminate in gangrene, yields, however, under the use of detergent lotions of vinegar, muriate of ammonia, and water.—*London Lancet*, Feb. 25, 1843.

